# Safe Introduction of Robotic Gastrectomy Facilitated by ICG-Guided Lymphography

**DOI:** 10.3390/jcm15124538

**Published:** 2026-06-11

**Authors:** Jure Salobir, Gašper Horvat, Blaž Trotovšek, Primož Sever

**Affiliations:** 1 Department of Abdominal Surgery, University Medical Center Ljubljana, 1000 Ljubljana, Slovenia; gasper.horvat@kclj.si (G.H.); blaz.trotovsek@kclj.si (B.T.); primoz.sever@kclj.si (P.S.); 2 Faculty of Medicine, University of Ljubljana, 1000 Ljubljana, Slovenia

**Keywords:** robotic gastrectomy, gastric cancer, indocyanine green, lymphography, learning curve, minimally invasive surgery

## Abstract

**Background/Objectives**: Robotic gastrectomy (RG) for gastric cancer requires structured implementation to ensure oncological safety, particularly in Western centers with lower case volumes. Indocyanine green (ICG)-guided near-infrared lymphography may facilitate adequate lymphadenectomy and reliable tumor localization. We report our stepwise institutional introduction of RG and evaluate the perioperative outcomes and diagnostic accuracy of ICG-guided lymphography. **Methods**: All consecutive patients who underwent curative-intent RG at the University Medical Center Ljubljana between June 2022 and September 2025 were retrospectively analyzed. The implementation followed a structured stepwise approach, beginning with subtotal gastrectomy and progressing to total gastrectomy after formal training at Severance Hospital, Yonsei University Health System, under the mentorship of Prof. Woo Jin Hyung. ICG was administered endoscopically the day before surgery for tumor localization and intraoperative lymphatic mapping. The operative learning curve was assessed by CUSUM analysis, segmented regression, and bootstrapped plateau estimation. **Results**: Thirty-eight patients underwent RG (17 subtotal and 21 total). R0 resection was achieved in 100% of cases. The conversion rate was 2.6%. Major complications (Clavien–Dindo ≥ IIIb) occurred in six patients (15.8%). The 30-day mortality rate was 0%, and the 90-day mortality rate was 2.6%. Bootstrapped plateau operative times were 321.2 min (95% Bias-corrected and accelerated confidence interval (BCa CI): 278.4–344.1) for subtotal and 413.5 min (95% BCa CI: 378.1–476.1) for total gastrectomy, with the steepest learning phase confined to the first 2–4 cases. ICG was used in 23 patients. In a validation subset of five patients (259 lymph node stations), the sensitivity and negative predictive value were both 100%, with zero false negatives in 57 ICG-negative stations. **Conclusions**: RG can be safely introduced using a structured, stepwise strategy supported by training at a high-volume expert center. ICG-guided lymphography demonstrated 100% sensitivity for tumor-draining nodal basins in a small validation cohort (*n* = 5), supporting the feasibility of the technique during program introduction and warranting prospective evaluation in larger series.

## 1. Introduction

Gastric cancer (GC) is the fifth most common malignancy and the fourth leading cause of cancer-related death worldwide. In Europe, its incidence is substantially lower than in East Asia; however, it retains considerable clinical importance, particularly because the majority of patients present with locally advanced disease. In Slovenia, approximately 130 patients undergo curative-intent GC surgery annually, the majority at two high-volume university centers, each performing approximately 44–55 GC surgeries annually [1].

Robotic gastrectomy (RG) has emerged as a technically advanced platform for minimally invasive GC surgery and has been pioneered and refined in high-volume Eastern centers [2]. Compared with conventional laparoscopic gastrectomy (LG), RG offers increased precision through motion scaling and tremor elimination, greater maneuverability via wristed instrument movement, and improved three-dimensional visualization. These advantages are associated with reduced intraoperative blood loss, a higher number of retrieved lymph nodes, and a potentially shorter learning curve [3,4,5,6]. The main limitations of RG are its high cost and long operative time. The absence of haptic feedback also poses a challenge for intraoperative tumor identification, necessitating preoperative lesion localization using endoscopic marking techniques. Submucosal injection of indocyanine green (ICG) provides an elegant solution: it demarcates the tumor margins under near-infrared (NIR) imaging and, as ICG diffuses through the gastric wall into regional lymphatic channels, simultaneously enables real-time fluorescence visualization of draining lymph node basins, thus combining tumor localization and intraoperative lymphatic mapping in a single maneuver [7].

The introduction of RG in Western centers is associated with specific challenges that are largely absent in East Asian high-volume settings. These include lower annual case volumes, a higher proportion of patients with elevated BMI, a greater prevalence of advanced-stage disease at diagnosis, and a higher proportion of proximal and gastroesophageal junction tumors [8]. Together, these factors increase operative complexity and may compromise oncological safety during implementation. Robust evidence of optimal implementation strategies for Western centers remains limited.

Previous studies have shown that a structured stepwise program with formal mentorship at a high-volume expert center can substantially shorten the learning curve and safeguard oncological outcomes during the introduction of RG [9]. Studies examining operative time trajectories during RG implementation suggest that 11–25 cases are typically required before performance stabilizes, although this figure is likely to be lower for surgeons with prior robotic experience in other subspecialties [10,11]. The role of ICG-guided lymphography in enhancing lymphadenectomy quality during RG has been demonstrated by the Severance Hospital group, where fluorescence-guided dissection improved lymph node retrieval and confirmed 100% sensitivity for metastatic nodal stations in a prospective cohort [7].

Herein, we report the stepwise institutional introduction of RG at the University Medical Centre Ljubljana, a high-volume center for GC surgery in Slovenia, by a surgeon experienced in both open GC surgery and robotic colorectal surgery. We describe our surgical technique, evaluate perioperative and oncological outcomes across the implementation period, characterize the operative learning curve using formal statistical methods, and assess the diagnostic accuracy of ICG-guided lymphography in a prospective validation cohort.

## 2. Materials and Methods

### 2.1. Study Design and Patient Selection

This retrospective observational study included all consecutive patients with histologically confirmed gastric adenocarcinoma who underwent curative-intent robotic gastrectomy at the University Medical Centre Ljubljana between 1 June 2022, and 17 September 2025. Patients were identified from a prospectively maintained institutional database. The inclusion criteria were: (1) histologically confirmed gastric adenocarcinoma; (2) elective, curative-intent (R0) surgical intent; and (3) robotic approach. Patients with synchronous distant metastases or peritoneal carcinomatosis detected at staging laparoscopy and those undergoing palliative procedures were excluded. All cases were discussed preoperatively at a dedicated multidisciplinary board (MDB) meeting attended by surgeons, oncologists, and radiation oncologists. Decisions regarding neoadjuvant chemotherapy, surgical extent, and reconstruction were made collectively. Patients were allocated to the robotic approach based on availability of the robotic system, no patient was excluded from the robotic approach on the basis of BMI, tumor stage, or anticipated technical complexity.

### 2.2. Stepwise Implementation Strategy

The robotic gastrectomy program was established using a structured, stepwise implementation strategy. The operating surgeon was experienced in both open GC and robotic colorectal surgeries before commencing the program. Robotic subtotal gastrectomy was first introduced on 1 June 2022. After the initial four subtotal gastrectomy cases, a structured clinical observership was completed at Severance Hospital, Yonsei University Health System, Seoul, Republic of Korea, under the direct mentorship of Professor Woo Jin Hyung. The observed technique was adopted in its entirety, including the use of ultrasonic shears as the primary energy device, linear-stapled lateral-to-lateral anastomotic technique, and systematic integration of near-infrared (NIR) fluorescence imaging. Robotic total gastrectomy was introduced following the training visit. After completing 38 cases, a second observership at the same institution was undertaken to further refine the technique and standardize the program.

### 2.3. Surgical Technique

Our approach follows that of Prof. Woo Jin Hyung and is described in detail elsewhere [12,13,14,15]. Here, we briefly describe our approach and the modifications made in the implementation of the procedure at our institution.

All procedures were performed using the da Vinci Xi^®^ Surgical System (Intuitive Surgical, Inc., Sunnyvale, CA, USA). The patients were positioned in a supine position with a 15° reverse Trendelenburg tilt. Pneumoperitoneum was established at 12–15 mmHg using a supraumbilical Veress needle. Six ports were placed: one 8 mm camera port at the supraumbilical position, two 8 mm robotic trocars in the right and left lateral abdominal positions, and two 12 mm ports positioned medially between the umbilical camera port and lateral robotic trocars. The left 12 mm trocar was positioned slightly cranially to facilitate the angulation of the robotic ultrasonic shears towards lymph node stations 6, 7, 8a, 9, and 11. The right 12 mm trocar was placed slightly caudally to facilitate duodenal stapling and construction of the esophago-jejunal anastomosis (EJA); during the resection phase, this port also served as the assistant trocar. A sixth AirSeal^®^ trocar (CONMED Corporation, Utica, NY, USA) was placed at the site of the planned Pfannenstiel incision to provide continuous insufflation via the AirSeal system and subsequently serve as the specimen extraction site. The robotic cart was docked on the patient’s left side. Liver retraction was achieved using a static suture-based sling technique [16].

Dissection was performed using ultrasonic shears as the primary energy device and Maryland bipolar fenestrated forceps for countertraction. Staging laparoscopy was first performed to exclude peritoneal or distant disease, followed by en bloc total omentectomy, infrapyloric dissection, and sequential lymphadenectomy from the infrapyloric region (station 6) to the suprapancreatic area (stations 7, 8a, 9, 11p, and 12a) and along the lesser curvature (stations 1 and 3). The extent of lymphadenectomy was determined based on preoperative clinical staging according to the Japanese Gastric Cancer Treatment Guidelines and patient-specific operative findings. In selected cases of total RG, station 11d was omitted from the dissection when the risk of vascular injury owing to difficult access to the distal splenic artery or dense peripancreatic tissue was deemed to outweigh the oncological benefit; this selective approach is consistent with the practice at European specialist centers and with our institutional open approach [17].

Multivisceral resection was performed when required for curative intent in the setting of direct organ invasion, as confirmed by intraoperative findings.

For subtotal gastrectomy, gastric transection was performed using a linear endostapler along the planned resection line, confirmed by ICG tumor marking when applicable. Reconstruction was performed as Roux-en-Y gastrojejunostomy or Omega-Braun reconstruction, using a linear-stapled lateral-to-lateral technique with the gastrojejunostomy created on the posterior gastric wall with a 60 mm linear stapler, and the common entry hole was closed with a running barbed suture. Enteroenterostomy was performed in a side-to-side manner in the same way. For total gastrectomy, the esophagus was divided using a linear stapler in an anterior-to-posterior direction after full mobilization of the gastroesophageal junction and dissection of the diaphragmatic crura. Reconstruction was performed as a Roux-en-Y esophagojejunostomy using a linear-stapled lateral-to-lateral technique on the posterior esophageal wall. The common entry hole was closed using a barbed suture. Jejunojejunostomy was performed 45–60 cm distal to the esophagojejunostomy. Concomitant cholecystectomy was selectively performed based on the presence of cholelithiasis, history of biliary symptoms, or patient-specific operative considerations. The specimen was retrieved through a 4–5 cm Pfannenstiel incision at the site of the insufflation trocar using a wound protector device.

### 2.4. ICG-Guided Tumor Localisation and Lymphatic Mapping

Near-infrared fluorescence imaging was incorporated from the outset using the Firefly^®^ NIR system (Intuitive Surgical, Inc., Sunnyvale, CA, USA) integrated into the da Vinci Xi platform. ICG (Verdye^®^, Diagnostic Green, Aschheim, Germany) was prepared as a 0.625 mg/mL solution and administered endoscopically the day prior to surgery via submucosal injection at four peritumoral quadrants (total dose 0.5 mg in 0.8 mL) [7,18]. ICG was used for two distinct purposes: (1) intraoperative tumor localization to guide the resection margin, particularly for lesions not identifiable under white light, and (2) lymphatic mapping to visualize sentinel and regional draining lymph node stations under NIR imaging (Firefly^®^ mode). The absence of residual fluorescent lymph nodes in the dissected field at the end of lymphadenectomy was confirmed using NIR imaging before closure.

In a validation subset of five patients, resected lymph node basins were separated ex vivo on the back-table and individually assessed with NIR fluorescence imaging (Firefly^®^). Each basin was classified according to its ICG fluorescence status and subsequent histopathological findings into four categories: ICG-positive with nodal metastasis (N+ICG+), ICG-positive without metastasis (N−ICG+), ICG-negative with nodal metastasis (N+ICG−), and ICG-negative without metastasis (N−ICG−).

### 2.5. Data Collection and Outcomes

Data were collected prospectively and verified retrospectively using electronic medical records. The recorded variables included age, sex, BMI, ASA physical status, ECOG performance status, neoadjuvant chemotherapy, tumor location and Siewert classification (for gastroesophageal junction tumors), type of gastrectomy, extent of lymphadenectomy, multivisceral resection, use of ICG, operative time, conversion to open surgery, type of reconstruction, cholecystectomy, R0 resection status, length of hospital stay, postoperative complications classified by the Clavien–Dindo system, 30-day and 90-day mortality, and histopathological results including Lauren classification, WHO subtype, and AJCC TNM pathological stage (8th edition).

The primary outcomes were R0 resection rate, major complication rate (Clavien–Dindo ≥ IIIb), 30-day mortality, and adequacy of lymphadenectomy. Secondary outcomes included operative time, blood loss, conversion rate, length of hospital stay, 90-day mortality, and diagnostic performance of ICG-guided lymphography (sensitivity, specificity, negative predictive value, positive predictive value, and false negative rate) in the validation subset.

### 2.6. Learning Curve Analysis

The operative learning curve was assessed using a complete chronological case series, analyzed separately for subtotal and total gastrectomy, using three complementary methods. Cumulative sum (CUSUM) analysis was applied to track cumulative deviations of individual operative times from the series mean; the CUSUM peak identifies the case after which the performance shifts from above to below the series mean. Segmented (piecewise) linear regression was used to identify statistically significant breakpoints in the operative time trajectory, providing an estimate of the inflection point with 95% confidence intervals (CIs). A five-case moving average was computed to smooth the case-by-case variability and visualize the overall trend. Bootstrapped plateau estimation was performed using the second half of each case series (bias-corrected and accelerated, BCa bootstrap, 10,000 iterations) to estimate stabilized operative times with 95% BCa confidence intervals. All statistical analyses were performed using R version 4.3 (R Foundation for Statistical Computing, Vienna, Austria).

The diagnostic accuracy of ICG-guided lymphography in the validation subset was assessed by computing the sensitivity, specificity, positive predictive value (PPV), and negative predictive value (NPV) at the lymph node station level, with 95% Wilson confidence intervals. The upper bound of the false-negative rate was estimated using the Clopper–Pearson exact binomial method and the rule-of-three approximation. Fisher’s exact test was used to assess the association between ICG fluorescence and histopathological nodal status. Between-group comparisons of lymph node yield (D2 vs. D1+ with station 12a) were performed using the Mann–Whitney U test, given the small sample size and non-normal distribution. Statistical significance was set at *p* < 0.05.

### 2.7. Use of Artificial Intelligence

During the preparation of this manuscript, the authors used AI-assisted writing tools (Claude Sonnet 4.6, Anthropic, San Francisco, CA, USA; ChatGPT, GPT-5, OpenAI, San Francisco, CA, USA) to support language editing, formatting consistency, and grammatical refinement of the text. AI tools were not used for study design, data collection, statistical analysis, interpretation of results, or generation of clinical conclusions. All AI-generated content was critically reviewed, edited, and validated by the authors, who take full responsibility for the accuracy and integrity of this publication.

## 3. Results

### 3.1. Patient Characteristics

Thirty-eight patients underwent curative-intent robotic gastrectomy for gastric adenocarcinoma during the study period. The mean age was 66.9 ± 12.5 years (median 69; range 41–89 years). Most patients were classified as ASA III (65.8%), followed by ASA II (31.6%) and ASA I (2.6%). Mean BMI was 27.5 ± 7.9 kg/m^2^. Eastern Cooperative Oncology Group (ECOG) performance score was 0 in 22 patients (57.9%), 1 in 11 patients (28.9%), and 2 in 5 patients (13.2%); no patient with ECOG performance score > 2 was operated on. All cases were discussed preoperatively at an MDB attended by surgeons and oncologists. Eleven patients (28.9%) received neoadjuvant chemotherapy based on the MDB recommendation.

The tumor was located in the lower third in 16 cases (42.1%), middle third in 11 cases (28.9%), upper third in four cases (10.5%), and cardia in four cases (10.5%), and involved the whole stomach in three cases (7.9%). Of the four cardia tumors, one was Siewert type I (2.6%), one was type II (2.6%), and two were type III (5.3%). According to the Lauren classification, 11 patients (29.0%) had intestinal-type, 10 (26.3%) diffuse-type, 10 (26.3%) mixed-type, and 7 (18.4%) unclassified adenocarcinoma. According to the WHO classification, 11 patients (29.0%) had the tubular subtype, 11 (29.0%) had the poorly cohesive subtype, 11 (29.0%) had the mixed type, and two (5.3%) had other subtypes; signet ring cells were present in 5 of the 11 poorly cohesive cases. No mucinous subtypes were observed.

Tumor stage was assessed according to the AJCC TNM classification. The T-stage distribution was as follows: pT1a in two patients (5.3%), pT1b in six (15.8%), pT2 in six (15.8%), pT3 in nine (23.7%), pT4a in 13 (34.2%), and pT4b in one (2.6%). In one patient who had previously undergone endoscopic submucosal dissection (ESD), no residual tumor was observed in the resection specimen. Nodal status was pN0 in 14 patients (36.8%), pN1 in 10 (26.3%), pN2 in seven (18.4%), pN3a in six (15.8%), and pN3b in one (2.6%). Final pathological staging was stage 0 in one patient (2.6%), IA in eight (21.1%), IB in one (2.6%), IIA in two (5.3%), IIB in 10 (26.3%), IIIA in nine (23.7%), IIIB in five (13.2%), IIIC in one (2.6%), and stage IV in one (2.6%).

### 3.2. Surgical Procedures and Intraoperative Outcomes

Robotic subtotal gastrectomy was performed in 17 (44.7%) patients, and robotic total gastrectomy was performed in 21 (55.3%) patients. Staging laparoscopy revealed no cases of peritoneal carcinomatosis or distant metastatic disease that precluded resection. Three patients required multivisceral resection due to local organ invasion: robotic total gastrectomy with wedge liver resection was performed in one patient, and robotic total gastrectomy with distal pancreatectomy and splenectomy was performed in another. One patient, in whom the tumor invaded the transverse colon, required conversion to open surgery—the only conversion in the series—yielding a conversion rate of 2.6%. Total omentectomy was performed in all patients. Concomitant cholecystectomy was performed in 28 patients (72.2%) based on the presence of cholelithiasis, a history of biliary symptoms, or patient-specific considerations.

In the subtotal gastrectomy group, lymphadenectomy was D1+ in five patients (29.4%) and D2 in 12 (70.6%). The mean operative time was 334.3 ± 57.5 min (median 327; range 229–417 min). In the total gastrectomy group, lymphadenectomy consisted of D1+ with additional station 12a dissection in 13 patients (61.9%) and D2 in eight (38.1%). The mean operative time was 412.9 ± 79.3 min (median 402; range 282–660 min). The difference in operative time between the two procedures was statistically significant (Wilcoxon rank-sum test, *p* = 0.0023), consistent with the greater extent of resection and complexity of reconstruction in total gastrectomy. R0 resection was achieved in 100% of patients. The mean intraoperative blood loss was 87.5 ± 40.4 mL (median 90; range 40–200 mL).

### 3.3. Postoperative Outcomes

Major complications (Clavien–Dindo ≥ IIIb) occurred in six patients (15.8%). Specific complications included anastomotic leakage of the esophagojejunal anastomosis in two patients (5.3%), anastomotic leakage of the gastrojejunal anastomosis in two patients (5.3%), biliary leakage from the duodenal stump in one patient (2.6%), intra-abdominal fluid collections or abscesses in five patients (13.2%), postoperative paralytic ileus (defined as absence of bowel movement beyond 48 h postoperatively) in three patients (7.9%), and obstructive ileus secondary to an internal hernia in one patient (2.6%). Five patients (13.2%) required reoperation, three (7.9%) were managed with endoscopic intervention, and two (5.3%) required percutaneous drainage by interventional radiology.

Postoperative bleeding occurred in seven patients (18.4%). Two cases of arterial bleeding were identified: one was managed with selective arterial embolization, while the other necessitated an emergency laparotomy with surgical hemostasis. The remaining five cases were self-limiting and were managed conservatively. Three patients developed subcutaneous emphysema of the abdominal and thoracic walls, a benign and self-resolving complication. The 30-day mortality was 0%, while the 90-day mortality was 2.6% (one patient). The median hospital stay was 9 days.

### 3.4. Learning Curve Analysis

#### 3.4.1. Subtotal Gastrectomy

**Figure 1:**
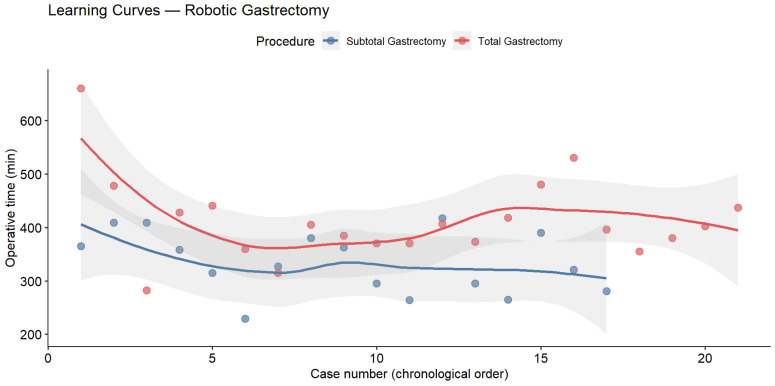
Combined learning curve for robotic subtotal and total gastrectomy. LOESS curves with 95% confidence bands are shown for each procedure, with individual case operative times connected by gray lines. Subtotal gastrectomy (*n* = 17): the LOESS curve descends gradually from approximately 400 min in the earliest cases to approximately 305 min by case 17, consistent with a continuous learning effect. Total gastrectomy (*n* = 21): the first case was an outlier (660 min); a steep early decline is followed by stabilization at approximately 380–420 min from case 6 onward. Bootstrapped plateau estimates: subtotal 321.2 min (95% BCa CI: 278.4–344.1 min); total 413.5 min (95% BCa CI: 378.1–476.1 min). Detailed CUSUM, segmented regression, and moving average analyses for each procedure are presented in Figures S1–S8 (Supplementary Materials).

The operative learning curve was assessed using CUSUM analysis, segmented regression, and bootstrapped plateau estimation on the complete chronological case series. The overall operative time trajectory was visualized using locally estimated scatterplot smoothing (LOESS) for each procedure. For subtotal gastrectomy (*n* = 17), CUSUM analysis peaked at case 4, indicating that the first four procedures were consistently performed above the series mean operative time; thereafter, a progressive decline was observed. The LOESS curve confirmed this gradual learning effect, descending from approximately 400 min in the earliest cases to approximately 305 min by case 17 (Figure 1). A five-case moving average revealed an initial operative plateau of approximately 340 min (cases 1–7), followed by a decline to approximately 300–310 min towards the end of the series. Segmented regression did not identify a statistically significant breakpoint, which is consistent with a gradual, continuous improvement pattern rather than an abrupt inflectional finding typical of small surgical series in which prior robotic and laparoscopic experience partially transfers to the new platform. Bootstrapped plateau estimation using the second half of the case series yielded a stabilized operative time of 321.2 min (95% BCa CI: 278.4–344.1 min) for subtotal gastrectomy (Figures S1–S4, Supplementary Materials).

#### 3.4.2. Total Gastrectomy

For total gastrectomy (*n* = 21), the CUSUM peak occurred at case 2, driven predominantly by the first case, which had an operative time of 660 min. Following this outlier, a rapid and sustained decline was observed, with the operative times stabilizing from case 6 onward. The LOESS curve demonstrated a steep early drop from the first-case outlier to a near-plateau at approximately 380–420 min, which was sustained through the remainder of the series (Figure 1). A secondary rise around cases 15–17 reflected a cluster of more complex procedures, but the trajectory returned to near the series mean by case 21. Segmented regression estimated a breakpoint at 2.6 cases (95% CI: 1.5–3.7), although formal statistical convergence was not achieved because of the high variability introduced by the initial outlier. The bootstrapped plateau operative time for total gastrectomy was 413.5 min (95% BCa CI: 378.1–476.1 min). The combined learning curve demonstrated that both procedures approached their respective plateaus within the observed case series, with the steepest learning phase for total gastrectomy confined to the first 2–3 cases (Figures S5–S8, Supplementary Materials).

### 3.5. Lymphadenectomy and ICG-Guided Lymphography

The median number of harvested lymph nodes was 35 (IQR 25.75–41.25). When stratified by procedure type, the median lymph node yield was 33 (IQR 26–39) for subtotal gastrectomy and 37 (IQR 27.50–41.50) for total gastrectomy. Within the total gastrectomy group, lymph node yield was significantly higher in patients who underwent D2 lymphadenectomy compared to those who underwent D1+ with additional station 12a dissection (median 41.5 (IQR 37.75–50) vs. 35 (IQR 25–39); *p*  =  0.042, Mann–Whitney U test). All patients achieved a minimum lymph node yield of 15, meeting the recommended threshold for adequate oncological staging.

ICG was administered preoperatively in 23 patients for tumor localization and intraoperative lymphatic visualization using near-infrared (NIR) fluorescence modality, with successful fluorescence mapping achieved in all cases. In a validation subset of five patients, resected lymph node basins were separated ex vivo and individually assessed using fluorescence imaging (Firefly^®^). A total of 259 lymph node stations were evaluated: 202 ICG-positive and 57 ICG-negative.

**Figure 2:**
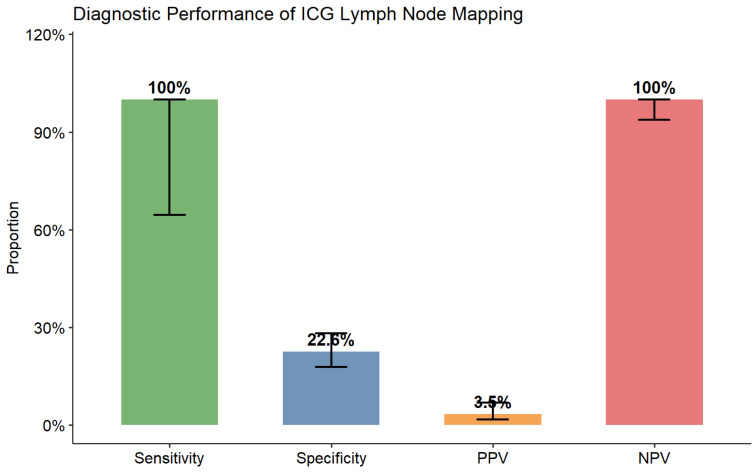
Diagnostic performance of ICG sentinel node mapping with 95% Wilson confidence intervals. The sensitivity and negative predictive value (NPV) were both 100%, confirming that no metastatic lymph node station was missed by ICG-guided lymphography. The specificity was 22.6%, and the positive predictive value (PPV) was 3.5%, reflecting the expected low prevalence of nodal metastasis in the overall station population.

Among the ICG-positive stations, seven harbored histopathologically confirmed nodal metastases (N+ICG+), and 195 were histologically negative (N−ICG+). Crucially, none of the 57 ICG-negative stations contained metastatic disease (N+ICG− = 0 and N−ICG− = 57). The diagnostic performance of ICG mapping demonstrated a sensitivity of 100% (95% CI: 64.6–100%), negative predictive value (NPV) of 100% (95% CI: 93.7–100%), and specificity of 22.6% (95% CI: 17.9–28.2%) (Figure 2). The positive predictive value (PPV) was 3.5% (95% CI: 1.7–7.0%), reflecting the low prevalence of metastasis (2.7%) in the overall population of lymph nodes. The observed false-negative rate was 0%, and the upper 95% confidence limit for the false-negative rate, estimated by the Clopper–Pearson exact binomial method, was 6.3% (rule of three approximation: 5.3%). Fisher’s exact test did not reach statistical significance (*p* = 0.353), likely reflecting the low prevalence of nodal metastasis and the high number of ICG-positive but histologically negative stations, rather than a failure of the mapping technique.

## 4. Discussion

Robotic gastrectomy has followed markedly divergent trajectories of adoption between Asia and Europe. In Japan, national health insurance coverage was granted for robotic gastrectomy in 2018, and the number of procedures has expanded rapidly. A nationwide registry reported 2295 robotic gastrectomies performed in 2019 alone, with the surgeon qualification rate reaching 98.9% and a 30-day mortality of 0.3–0.6% [19]. In South Korea, robotic gastrectomy has been adopted broadly by laparoscopic surgeons since 2005, with major centers reporting consecutive series exceeding 1000 procedures and contributing a large body of evidence on oncological equivalence, learning curves, and intraoperative technique [2,10,20,21]. Cost remains a relevant difference between the two systems, as the Korean National Health Insurance does not reimburse robotic platform use, with costs transferred to patients or private insurers [22]. In Europe, by contrast, adoption has remained limited, heterogeneous, and concentrated in tertiary referral centers. A European perspective review noted that robotic gastrectomy has not achieved significant traction in Europe and that most published series come from Asia, with Western cohorts differing systematically in tumor stage, tumor location, and patient BMI [8]. A pan-European survey of 1045 surgeons across 38 countries identified inconsistent training pathways, limited robotic access, and absence of standardized curricula as the principal barriers to wider adoption of robotic gastrointestinal surgery in Europe [23].

The introduction of new surgical techniques requires careful, stepwise implementation to maintain patient safety [24]. In robotic gastrectomy, a stepwise program supported by formal mentorship at a high-volume Eastern center, combined with prior experience in robotic surgery in other subspecialties, can compress the learning trajectory and reduce the risk of implementation-phase complications.

As a novel approach, robotic gastrectomy is subject to variability between centers. A key source of technical variability in robotic total gastrectomy is the method of esophagojejunal anastomosis (EJA) construction [4]. Three techniques are currently popular among surgeons. Hand-sewn EJA is employed at several centers, where the robotic platform confers a distinct advantage over conventional laparoscopy for intracorporeal suturing. The leakage rate for hand-sewn EJA was reported to be 8–9% [25,26]. Somewhat lower EJA leakage rates (6%) have been reported with linear stapled anastomosis [26]. This is consistent with our experience of a 5.3% EJA leakage rate.

Some centers utilize robot-assisted gastrectomy with a circular stapler for EJA formation. In a multicenter study, this was associated with a leakage rate of 21% [26]. This difference was not observed in a large retrospective study with similar leakage rates in the linear (1.9%) and circular (2.1%) stapling groups; however, the circular stapled anastomosis was associated with a higher incidence of anastomotic stenosis (10.8% vs. 0.5%, *p* < 0.001) [27].

Another source of technical variability is the choice of energy device. In Japan, a double-bipolar method combining Maryland and fenestrated bipolar forceps (both Intuitive Surgical, Sunnyvale, CA, USA) driven by an external energy platform is widely used, permitting independent settings for tissue division and soft coagulation [28]. At our institution, we perform the majority of the dissection using the left-hand ultrasonic shears approach, as described by Hyung [29]. Compared with the double-bipolar technique, this has been associated with a modestly shorter operative time and lower blood loss, although the absolute differences (approximately 22 min and 10 mL) are of limited clinical significance [30]. The principal limitation of ultrasonic shears is instrument rigidity, which precludes wristed movements; in our practice, this is mitigated by the precise placement of the left middle trocar in line with the principal dissection planes. Additional integrated energy devices are available within the da Vinci ecosystem, including Vessel Sealer and SynchroSeal (both Intuitive Surgical). However, in our experience, the improved articulation of these instruments did not translate into a practical advantage over ultrasonic shears for lymphadenectomy, as the blunter tip geometry reduced the precision of fine dissection.

Dissection in RG can be further facilitated using preoperative endoscopic ICG injection. The technique, established at Severance Hospital by Kwon et al., demonstrated that fluorescent lymphography-guided lymphadenectomy yielded 95.3% sensitivity for detecting all metastatic lymph node stations and an NPV of 99.3% per station in 592 patients who underwent minimally invasive gastrectomy [7]. Subsequent studies have confirmed these findings in specific clinical scenarios. In GC patients undergoing RG after prior endoscopic submucosal dissection, station-level sensitivity and NPV were 100% [31]. In advanced disease, a false-negative rate of 4.7% was reported, with advanced T-stage and lymphovascular invasion as independent predictors of false-negative results [18]. Lee et al. further demonstrated that fluorescent lymphography significantly increased lymph node yield at the splenic hilum during total gastrectomy, with a station-level NPV of 97.1% at this anatomically challenging location [32]. ICG-guided robotic surgery has also been identified as a significant independent predictor of achieving proper lymphadenectomy (odds ratio 3.15) [33].

While adequate nodal yield can be achieved through standard lymphadenectomy, this combined evidence shows the principal value of ICG lymphography which, in our experience, lies in three complementary domains: real-time tumor localization, especially important in the absence of tactile feedback, identification of aberrant drainage patterns and intraoperative quality assurance through confirmation of a fluorescence-free operative field.

Besides improved lymphatic yield, recent large-scale evidence points towards a prognostic role of ICG lymphography in improving overall recurrence rate and overall survival [34,35].

We incorporated preoperative ICG injection as a routine practice for all tumors smaller than 5 cm. In our validation subset of five patients across 259 lymph node stations, ICG-guided lymphography achieved 100% sensitivity and 100% NPV, with zero false negatives among 57 ICG-negative stations. The 95% Clopper–Pearson upper bound for our false-negative rate is 6.3%, consistent with the 4.7% reported by Kwon et al. [7]. The low specificity (22.6%) and PPV (3.5%) reflect the low prevalence of nodal metastasis in the study population and are a structural feature of fluorescent mapping rather than a limitation. The median lymph node yield was 35 (IQR 25.75–41.25) overall—33 for subtotal and 37 for total gastrectomy—with ≥15 nodes achieved in all cases, consistent with European robotic and laparoscopic series reporting median yields of 26–37 nodes [26,36]. These results should be interpreted with caution given the small size of the validation cohort (*n* = 5), which provides only a preliminary signal of feasibility and is insufficient to draw definitive conclusions regarding diagnostic performance.

Of note, our protocol was designed as a fluorescence-guided lymphatic mapping technique rather than a formal sentinel node navigation strategy: ICG was used to visualize the complete draining nodal basin and to confirm the completeness of lymphadenectomy rather than to identify a discrete sentinel node and modify the extent of dissection accordingly. The ICG injection protocol (0.625 mg/mL solution, 0.8 mL total dose, submucosal injection at four peritumoral quadrants the day prior to surgery) was adopted directly from the validated protocol established at Severance Hospital, where the lead author completed formal training as part of the structured implementation program [7]. The optimal dose and timing of ICG injection remain incompletely standardized in the literature [37]. In our view, consistency with an established high-volume protocol is particularly important to ensure reproducible imaging quality and meaningful comparison with reference series.

The 16–24 h preoperative window allows adequate migration of ICG from the submucosa into the regional lymphatic network while maintaining sufficient fluorescence intensity at the time of surgery. This timing and dose were associated with reliable fluorescence in all 23 patients in whom ICG was used in our series, with no adverse events related to ICG administration.

A recognized advantage of RG is the quicker adaptation by experienced GC surgeons. Studies examining the learning curve achieved by surgeons introducing RG suggest that 11–25 cases are required during the implementation phase [38,39,40].

In our series, the steepest portion of the learning curve, as identified by CUSUM analysis, was confined to the first 2–4 cases for both procedure types; this reflects the initial adaptation phase rather than a formal plateau. Segmented regression did not identify a statistically significant inflection point for subtotal gastrectomy, which is consistent with a pattern of continuous, gradual improvement typical of surgeons who transfer substantial prior robotic and laparoscopic experience to a new procedure [38,39,40]. For total gastrectomy, the segmented regression identified a breakpoint, though the small sample size warrants cautious interpretation. Bootstrapped LOESS plateau estimates of 321.2 min for subtotal gastrectomy and 413.5 min for total gastrectomy reflect stabilization of operative time rather than technical mastery, and should not be interpreted as evidence that the learning curve was complete. The subtotal plateau compares favorably with the mean of 361 min reported by Uyama et al. during early program implementation [38]. Direct comparison is however limited by differences in case volume, patient selection, and institutional context. Prior experience in robotic colorectal surgery likely contributed to a shortened initial adaptation period, facilitating earlier instrument handling proficiency and port placement confidence. These findings support the view that a structured stepwise program with formal training at a high-volume center can compress the initial adaptation phase, though a larger case series will be required to fully characterize the learning trajectory. The longer operative times consistently observed in Western high-volume centers compared with Eastern centers have several contributing factors beyond the learning curve itself. In our series, total omentectomy was performed in all cases, and concomitant cholecystectomy was performed in 72.2% of patients. Although cholecystectomy does not require cart re-docking and uses the same instrument configuration as gastrectomy, it nonetheless extends the overall operative time [41]. The higher BMI and greater proportion of advanced-stage and proximal tumors in Western patients also contribute to longer dissection and reconstruction times compared with the predominantly early-stage, lower-third cohorts in Eastern high-volume series. In our cohort, major complications (Clavien–Dindo ≥ IIIb) occurred in 15.8% of patients, and the overall anastomotic leakage rate was 10.6%, with a 90-day mortality of 2.6% and a median hospital stay of 9 days. These outcomes fall within the range reported in European series, where major complications occur in 12.2–18%, 90-day mortality ranges from 2.5 to 9.6%, anastomotic leakage occurs at rates from 8.7 to 10%, and median hospital stay is between 8.5 and 9 days [7,24,26,27,28,42]. In the subtotal gastrectomy subgroup, the linear-stapled anastomotic leakage rate of 5.3% closely approximates the 3% reported in the international UGIRA multicenter registry of 759 robotic gastrectomies [26]. By contrast, high-volume Asian and North American expert centers consistently report substantially lower complication burdens. The Memorial Sloan Kettering experience in 220 robotic gastrectomies reported only 7% major complications, a 2% anastomotic leak rate, and 0.9% mortality [43]. A Yonsei analysis of 5839 gastrectomies (including 436 robotic cases) documented an overall complication rate of 10.5% with 0.4% mortality [44]. After total gastrectomy with Roux-en-Y reconstruction, the global UGIRA cohort reported anastomotic leakage rates of 6–8% depending on the anastomotic technique [26]. Importantly, a multicenter Korean prospective study of 502 robotic gastrectomies demonstrated that approximately 25 cases are required to overcome the complication-related learning curve, with moderate complication rates declining from 20% during the first 25 cases to 6.4% beyond case 89 [10]. The higher complication profile observed in our series is likely attributable to several cohort-specific factors: the early implementation phase, a high proportion of total gastrectomies (55.3%), three multivisceral resections (including a combined distal pancreatectomy and splenectomy), consecutive inclusion of unselected patients regardless of BMI or tumor stage, and a higher proportion of locally advanced disease (pT3–4 in 60.5%) than typically reported in Asian series, where early gastric cancer predominates. These rates are consistent with the early phase of a Western robotic gastrectomy program and are expected to improve with increasing case volume, in line with the learning curves documented in larger series.

This study had several limitations that should be considered when interpreting the findings. Its retrospective, single-center design limits its generalizability and introduces selection bias. The sample size of 38 cases, while sufficient to characterize the early institutional learning curve, precludes definitive conclusions about long-term oncological outcomes; survival data are not yet mature enough. The ICG validation subset of five patients, although analyzed at high station-level resolution (259 stations), was too small to provide precise point estimates of the false-negative rate; our results should be interpreted as hypothesis-generating and confirmed in a larger prospective cohort. We also could not formally account for all patient-level confounders, including comorbidity burden and nutritional status, which may influence perioperative outcomes independently of the surgical approach. The same applies to the learning curve analysis, which is inherently dependent on operative time as a surrogate for performance and does not capture all dimensions of surgical quality, such as lymph node yield, conversion risk, or long-term oncological control. A notable limitation of this study is the absence of a concurrent laparoscopic or open gastrectomy control group from our institution. While the oncological and perioperative outcomes of robotic gastrectomy have been established in large Asian series and meta-analyses, a meaningful comparison with our own institutional laparoscopic or open experience would have been methodologically more rigorous. However, such a comparison is inherently problematic in the context of program introduction: early robotic outcomes necessarily reflect a learning phase and do not represent the mature performance of the program, making direct comparison with established techniques potentially misleading rather than informative. Furthermore, any observed differences during this phase would be difficult to attribute to the robotic platform itself rather than to the novelty of the approach. Ideally, the comparative efficacy and safety of robotic versus laparoscopic gastrectomy should be evaluated in a prospective, randomized controlled trial setting.

## 5. Conclusions

Robotic gastrectomy can be safely implemented using a structured, stepwise strategy led by an experienced GC surgeon and supported by formal training at a high-volume expert center. ICG-guided lymphography demonstrated promising diagnostic performance in a small validation cohort, supporting its role in tumor localization and nodal basin identification during program introduction; prospective evaluation in larger series is warranted. Acceptable morbidity, low conversion rate, R0 resection in all patients, and adequate oncologic lymphadenectomy were achieved during the implementation.

## Data Availability

The data presented in this study are available on request from the corresponding author due to ethical and privacy restrictions. The data are not publicly available because they contain information that could compromise the privacy of research participants and are subject to institutional and regulatory restrictions.

## Supplementary Materials

The following supporting information can be downloaded at: https://www.mdpi.com/article/10.3390/jcm15124538/s1. Figure S1: Robotic subtotal gastrectomy: operative time by case number with LOESS. Figure S2: CUSUM chart for robotic subtotal gastrectomy. Figure S3: Segmented regression for robotic subtotal gastrectomy. Figure S4: Five-case moving average for robotic subtotal gastrectomy. Figure S5: Robotic total gastrectomy: operative time by case number with LOESS. Figure S6: CUSUM chart for robotic total gastrectomy. Figure S7: Segmented regression for robotic total gastrectomy. Figure S8: Five-case moving average for robotic total gastrectomy.

## Abbreviations

The following abbreviations are used in this manuscript:

AJCC	American Joint Committee on Cancer
ASA	American Society of Anesthesiologists
BCa CI	Bias-corrected and accelerated confidence interval
BMI	Body mass index
CI	Confidence interval
CUSUM	Cumulative sum
ECOG	Eastern Cooperative Oncology Group
EJA	Esophagojejunal anastomosis
ESD	Endoscopic submucosal dissection
GC	Gastric cancer
ICG	Indocyanine green
LG	Laparoscopic gastrectomy
LOESS	Locally estimated scatterplot smoothing
MDB	Multidisciplinary board
NIR	Near-infrared
NPV	Negative predictive value
OR	Odds ratio
PPV	Positive predictive value
RG	Robotic gastrectomy
TNM	Tumor–node–metastasis
WHO	World Health Organization
