# Supplementary material for: Safe Introduction of Robotic Gastrectomy Facilitated by ICG-Guided Lymphography
Source: J Clin Med. 2026 Jun 11;15(12):4538. doi: 10.3390/jcm15124538 (PMC13302083; doi:10.3390/jcm15124538)
Supplement: Supplementary file 1 [file jcm-15-04538-s001.zip › jcm-4308526-supplementary.pdf]

Supplementary Materials

Safe Introduction of Robotic Gastrectomy Facilitated by ICG-Guided Lymphography

Jure Salobir, Gašper Horvat and Primož Sever

Figures S1–S4 show the detailed learning curve analyses for robotic subtotal gastrectomy; Figures S5–S8 show the corresponding analyses for robotic total gastrectomy. Each set comprises, in order: the operative time trajectory with LOESS smoothing, the CUSUM chart, the segmented regression, and the five-case moving average.

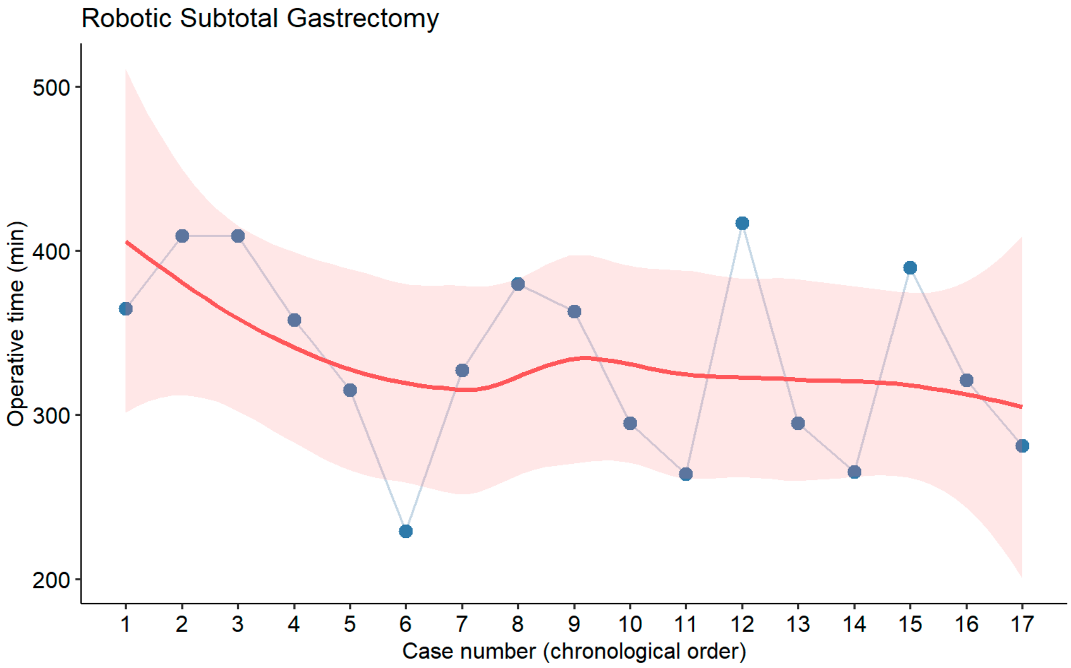

Figure S1. Robotic subtotal gastrectomy: operative time by case number with LOESS smoothing (red curve, 95 % CI shaded). Individual case times are connected by grey lines. The LOESS curve descends from approximately 400 min in the first cases to approximately 305 min by case 17, confirming a gradual learning effect.

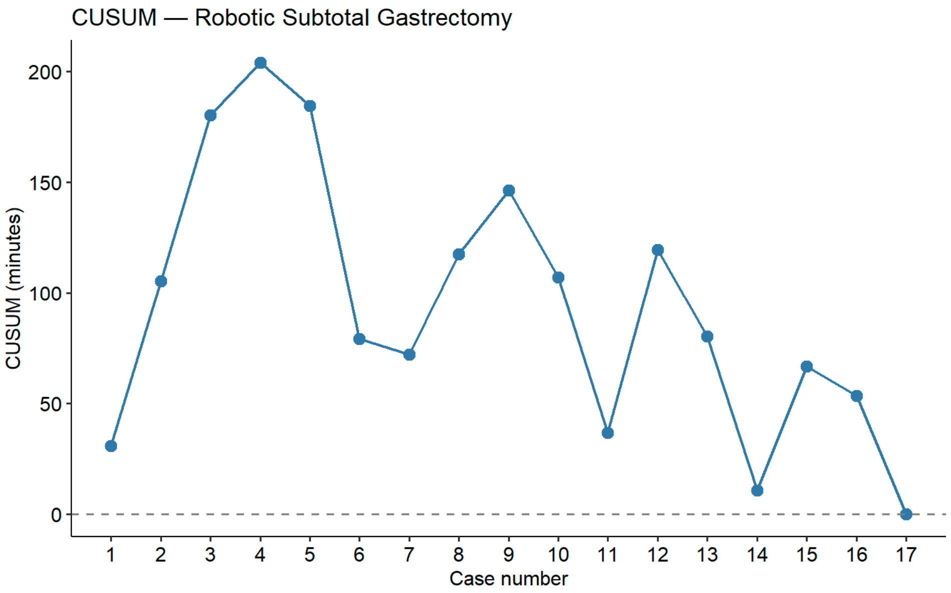

Figure S2. CUSUM chart for robotic subtotal gastrectomy. The curve peaks at case 4, after which cumulative operative times fall progressively below the series mean, indicating sustained improvement in operative efficiency throughout the series.

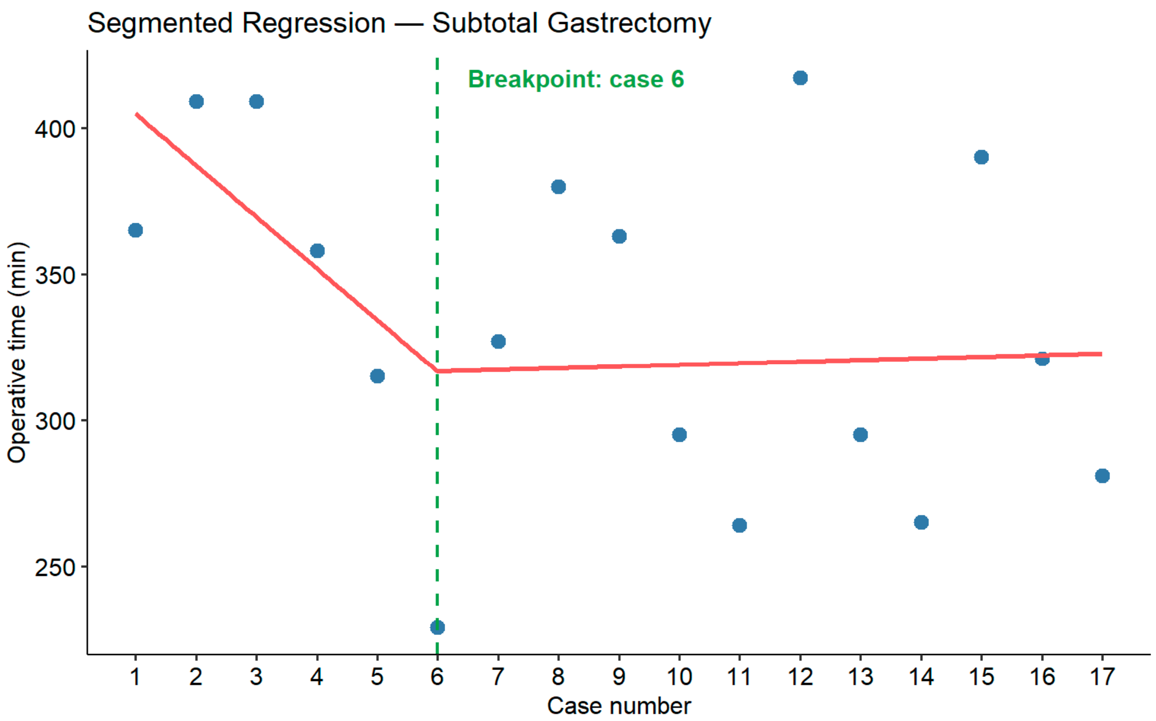

Figure S3. Segmented regression for robotic subtotal gastrectomy. Estimated breakpoint at case 6 (95 % CI: −1.9–13.9). The overall negative slope confirms a continuous learning effect; the absence of a statistically significant inflection point is consistent with the small sample size and gradual improvement pattern.

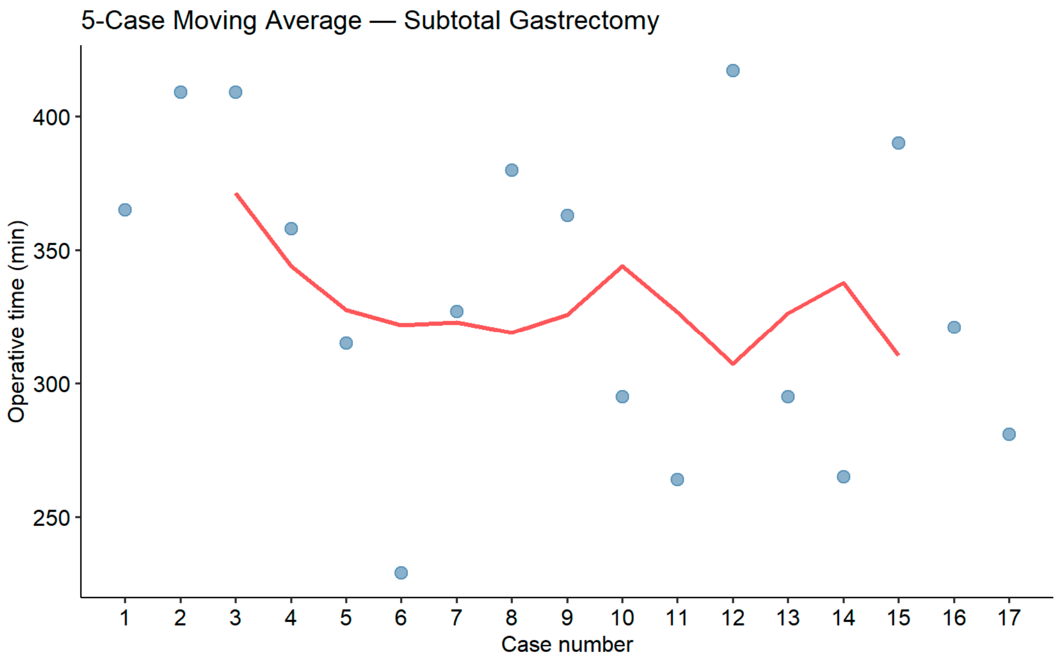

Figure S4. Five-case moving average for robotic subtotal gastrectomy. An initial plateau of approximately 340 min (cases 1–7) is followed by a progressive decline to 300–310 min, reflecting stabilisation of operative performance toward the end of the series.

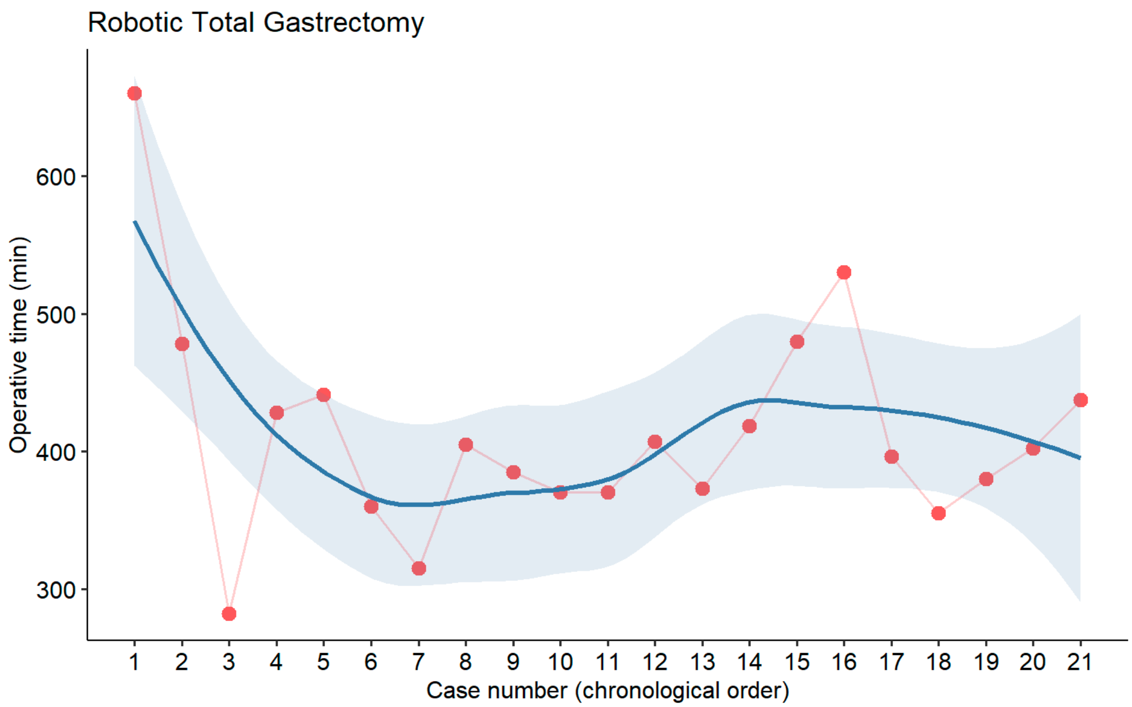

Figure S5. Robotic total gastrectomy: operative time by case number with LOESS smoothing (red curve, 95 % CI shaded). The first case (660 min) represents an outlier; a steep early decline is followed by stabilisation from case 6 onward at approximately 380–420 min.

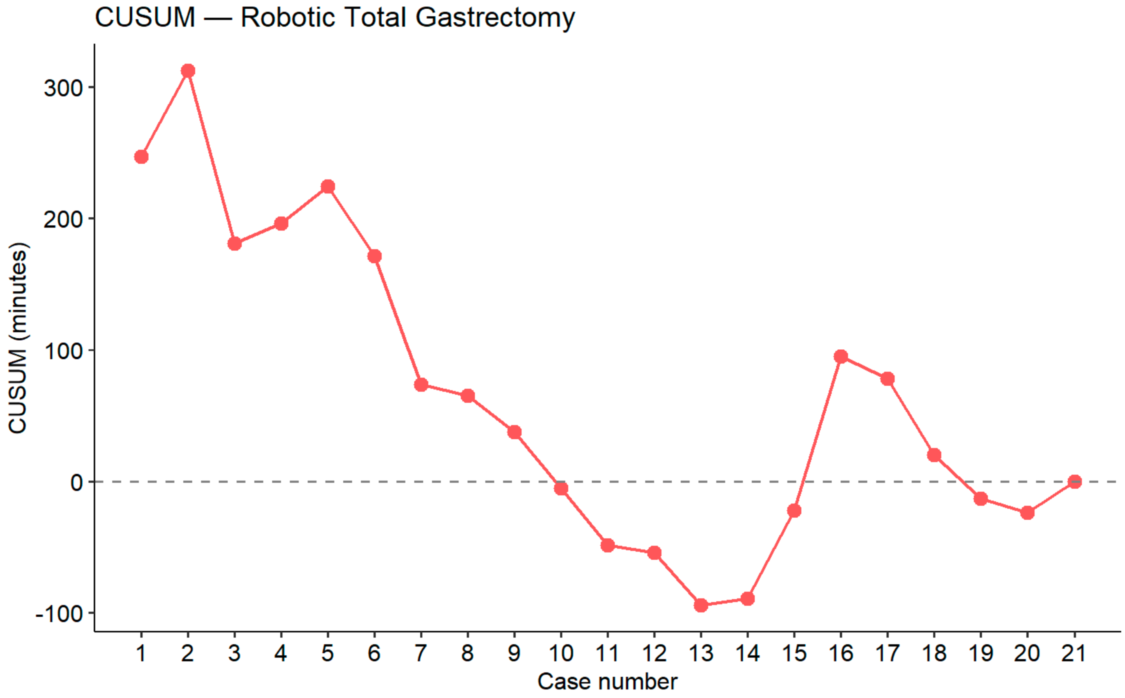

Figure S6. CUSUM chart for robotic total gastrectomy. The curve peaks at case 2, driven by the prolonged first case. The steep sustained decline through case 13 reflects rapid early improvement; a secondary rise around cases 15–17 corresponds to a cluster of more complex procedures.

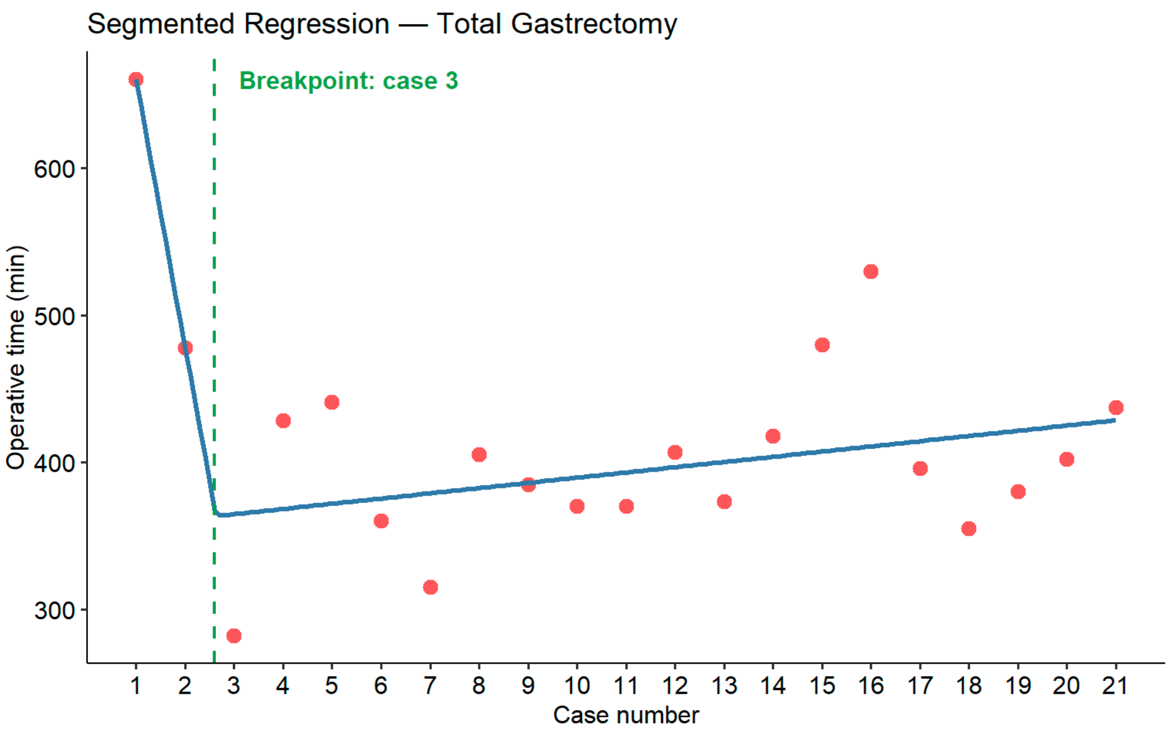

Figure S7. Segmented regression for robotic total gastrectomy. Estimated breakpoint at case 2.6 (95 % CI: 1.5–3.7). The steep initial descent followed by a near-horizontal plateau is characteristic of a learning curve dominated by rapid early improvement.

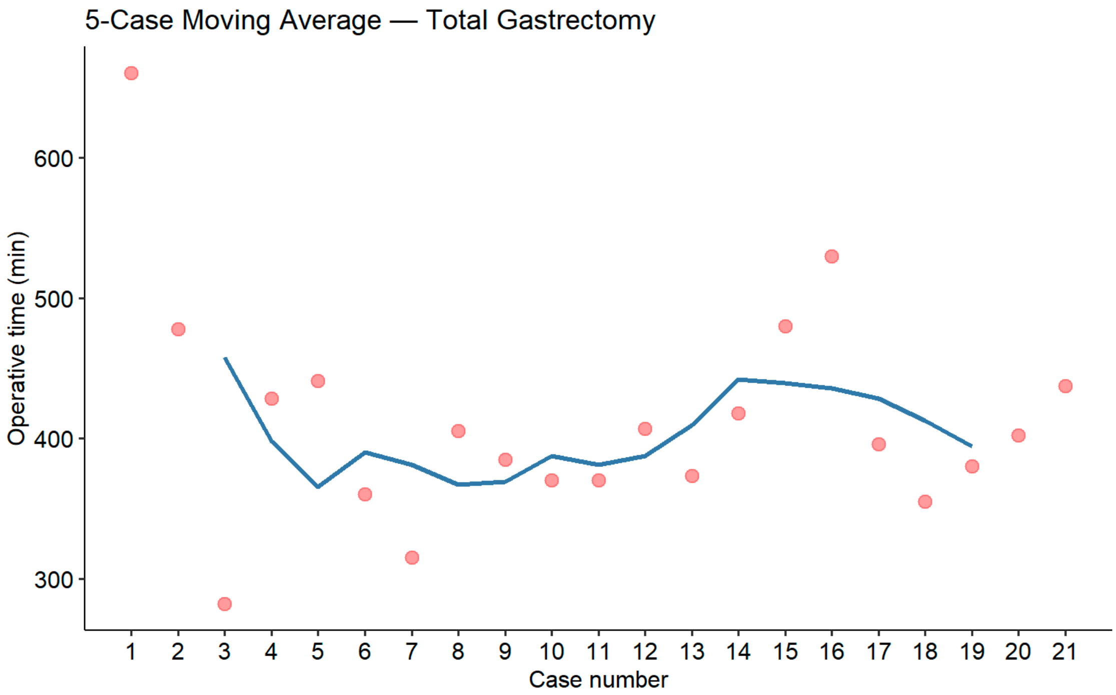

Figure S8. Five-case moving average for robotic total gastrectomy. A rapid decline from approximately 460 min to 380 min occurs within the first 5–6 cases, followed by relative stabilisation. A secondary peak around cases 14–16 reflects procedure-complexity clustering before returning to approximately 400 min.
